# Changes in Toxin Quantities Following Experimental Manipulation of Toxin Reserves in *Bufo bufo* Tadpoles

**DOI:** 10.1007/s10886-019-01045-9

**Published:** 2019-01-26

**Authors:** Zoltán Tóth, Anikó Kurali, Ágnes M. Móricz, Attila Hettyey

**Affiliations:** 1 Lendület Evolutionary Ecology Research Group, Plant Protection Institute, Centre for Agricultural Research, Hungarian Academy of Sciences, Herman Ottó Str. 15, Budapest, H-1022 Hungary; 2 Department of Pathophysiology, Plant Protection Institute, Centre for Agricultural Research, Hungarian Academy of Sciences, Herman Ottó Str. 15, Budapest, H-1022 Hungary

**Keywords:** Bufadienolides, Toxin production, Chemical defence, Hormonal treatment, Stress response, Tadpoles

## Abstract

Possessing toxins can contribute to an efficient defence against various threats in nature. However, we generally know little about the energy- and time-demands of developing toxicity in animals, which determines the efficiency of chemical defence and its trade-off with other risk-induced phenotypic responses. In this study we examined how immersion into norepinephrine solution inducing the release of stored toxins, administration of mild stress mimicking predator attack or simple handling during experimental procedure affected the quantity and number of toxin compounds present in common toad (*Bufo bufo*) tadpoles as compared to undisturbed control individuals, and investigated how fast toxin reserves were restored. We found that total bufadienolide quantity (TBQ) significantly decreased only in the norepinephrine treatment group immediately after treatment compared to the control, but this difference disappeared after 12 h; there were no consistent differences in TBQ between treatments at later samplings. Interestingly, in the norepinephrine treatment approximately half of the compounds characterized by >700 m/z values showed the same changes in time as TBQ, but several bufadienolides characterized by <600 m/z values showed the opposite pattern: they were present in higher quantities immediately after treatment. The number of bufadienolide compounds was not affected by any treatments, but was positively related to TBQ. Our study represents the first experimental evidence that toxin quantities returned to the original level following induced toxin release within a very short period of time in common toad tadpoles and provide additional insights into the physiological background of chemical defence in this model vertebrate species.

**Electronic supplementary material:**

The online version of this article (10.1007/s10886-019-01045-9) contains supplementary material, which is available to authorized users.

## Introduction

Many organisms are known to use toxins to defend themselves against naturally occurring threats (Chen 2008; Kempken and Rohlfs 2010; Kicklighter 2012). If adaptive plasticity evolves in such chemical defences, the presence of pathogens/parasites, predators or competitors can induce a physiological response that results in enhanced toxin production (manifested either in the increased number of toxins or in higher amounts of effective compounds), leading to better chances of survival in more risky environments (Harvell 1990; West-Eberhard 1989). Plasticity in chemical defence is well known in plants (Ahuja et al. 2012; Mithöfer and Boland 2012; War et al. 2012), and previous studies have also shown that in several animal species individuals can adjust their defensive toxin production in response to the risk of predation, competition or infection (reviewed in Hettyey et al. 2014). However, unlike in many plants (Ali and Agrawal 2012; Gonzales-Vigil et al. 2011) and (cyano)bacteria (Dittmann et al. 2013; Wang et al. 2016), the biosynthetic pathways and the enzymes involved in the production of toxins are often unknown in animals (Bane et al. 2014; Mebs 2001, but see McGugan et al. 2016). Yet investigations on the proximate mechanisms of toxin production are important because physiological constraints and limits of toxicity may fundamentally influence the effectiveness of toxins and affect its trade-offs with other forms of inducible defence, such as plastic responses in morphology, development or behaviour.

In species where *de novo* biosynthesis takes place through a complex metabolic pathway and specific enzymes are involved in toxin production (i.e. toxic compounds are not produced by symbiotic bacteria or uptaken from the diet), physiological constraints are simply definable. Such constraints may be the time, energy and precursor availability that are required for toxin synthesis and, thus, for the build-up of baseline toxicity, for the replenishment of depleted toxin reserves and for the production of an increased quantity of toxins if induced by environmental cues. For instance, Richelle-Maurer et al. (2003) found that in the sponge *Agelas conifera* individuals exhibited a three- to four-fold rise in levels of endogenous bromopyrrole alkaloids in response to simulated predator attacks compared to control conditions. More interestingly, one of the two predominant compounds’ concentration increased 12 h after the inflicted damage, whereas the increase in the other compound’s concentration was delayed by six days. This finding implies that different compounds within a toxic blend may have different production costs or time requirements, which may also substantially affect their function or deterrence efficiency. However, in most species physiological constraints related to toxin production were rarely studied, although these are especially relevant in species where toxins are excreted during antagonistic encounters and, thus, their reserves need to be restored.

Amphibians are popular model organisms for the study of various aspects of inducible responses, including chemical defence (Mangoni et al. 2001; Toledo and Jared 1995). Most bufonid species produce bufadienolides (Hayes et al. 2009; Mebs et al. 2007; Sciani et al. 2013), cardiotoxic steroids that inhibit Na^+^/K^+^-ATPases (Steyn and van Heerden 1998) and make these animals more or less unpalatable to most vertebrate predators (Gunzburger and Travis 2005). In toads, more than 100 different bufadienolide compounds have been identified so far, some of which may be the result of *in situ* bacterial biotransformation (Hayes et al. 2009). The biosynthesis of bufadienolides starts with cholesterol, but the intermediate compounds and associated enzymes along the biosynthetic pathway are not yet known, although a novel “acidic” bile acid pathway has been proposed to be involved in the synthesis of marinobufagenin, an endogenous Na^+^/K^+^-ATPase inhibitor also present in mammals (Fedorova et al. 2015). Bufadienolide compounds are usually classified as either free type bufogenins or conjugated type bufotoxins (although a “bufolipin” sub-class has also been identified in cane toad eggs and ovaries; Crossland et al. 2012), according to the esterification of the C-3 hydroxyl group of the steroid nucleus (Rodríguez et al. 2017). While bufogenins possess a free hydroxyl group at C-3, bufotoxins are typified by the conjugation to this ligand to form various esters (Wang et al. 2011), which generally results in a detectable increase in their mass-to-charge ratio (m/z value); however, sulphate conjugates can have m/z values similar to that of the bufogenins (Meng et al. 2016). Previous studies, which investigated the structure-activity relationship in bufadienolide compounds, found that bufogenins are generally more potent than bufotoxins (Kamano et al. 1998; Lee et al. 1994; Meng et al. 2016; Shimada et al. 1987a), although some bufotoxins containing a suberoyl-arginine group in their side chain are more toxic than their respective bufogenin analogues (Shimada et al. 1985, 1986, 1987b). It has been proposed that an increasing structural diversity of bufadienolides could be advantageous in terms of survival if it enhances the probability of interfering with a wider subset of Na^+^/K^+^-ATPase isoforms (Hayes et al. 2009), however we still do not know how these compounds are related to each other in the bufadienolide biosynthetic pathway, and whether or not there are any functional differences between various compounds or variation in physiological limits related to their production.

In this study, we used common toad (*Bufo bufo*) tadpoles to examine how fast toxin quantities can be restored after experimentally induced toxin release. During this experiment we manipulated tadpoles’ bufadienolide reserves by either immersing them into a norepinephrine hormonal solution or by applying mild, but abrupt mechanical disturbance. Norepinephrine has been successfully applied in previous studies to induce toxin release from skin glands in tadpoles of several species (e.g. Calhoun et al. 2016), including the common toad (Kurali et al. 2016). We predicted that immersion into norepinephrine solution would lead to a decrease in the amount of bufadienolides in tadpoles’ toxin reserves due to enforced excretion, but expected bufadienolide reserves to become similar to that of untreated individuals within five days. Furthermore, if a mild, non-invasive stressor can also trigger active toxin release, the amount of detectable bufadienolides in tadpoles would also decrease, but to a lower extent, so that the induced difference between treated and untreated individuals would diminish more rapidly. Tadpoles kept in control conditions were expected to maintain a relatively constant level of toxicity throughout the experiment. The number of bufadienolide compounds was predicted to remain unaffected by the applied treatments as these do not hinder the production pathway of bufadienolides in any way according to our present knowledge.

## Methods and Materials

### Study Species

The common toad (*Bufo bufo* Linnaeus, 1758) is an anuran amphibian that is widespread across Europe (Gasc et al. 1997) and uses various types of waterbodies for breeding. Due to the high environmental variability of these aquatic habitats, offspring may be exposed to widely varying abundances of predators, competitors and pathogens during larval ontogeny (Bókony et al. 2016; Ujszegi et al. 2017). Common toad tadpoles have previously been found to exhibit plasticity in behaviour (Marquis et al. 2004; Nunes et al. 2013, but see Richter-Boix et al. 2007), life-history (e.g., Lardner 2000; Laurila et al. 1998; Nunes et al. 2014), and morphology (Nunes et al. 2014; Van Buskirk 2009), although the extent/intensity of these plastic responses is relatively weak compared to those of ranid tadpoles (e.g. Lardner 2000; Laurila et al. 1998). As with most bufonid species, common toads produce cardiotoxic steroids, called bufadienolides, *de novo*, and we have recently shown that tadpoles are capable of synthesizing their own bufadienolides (Üveges et al. 2017). Histological and ultrastructural studies on this species have also demonstrated that underlying secretory cells are already present in larvae and that they are not distributed evenly in the skin especially at earlier developmental stages (Delfino et al. 1995; Kulzer 1954). Secretions from these cells can also reach the exterior of tadpoles’ skin in bufonids, including this species (Kulzer 1954; Le Quang Trong 1973; Meyer 1962). Recent studies investigating various aspects of *B. bufo* tadpoles’ bufadienolide production have also revealed that individuals can respond plastically in the amount of produced bufadienolides to the perceived intensity of competition (Bókony et al. 2018) and to the presence of a glyphosate-based herbicide (Bókony et al. 2017). Moreover, experimentally induced toxin release was found not to be associated with high costs measured in several fitness-related traits (Kurali et al. 2016).

### Animal Collection and Husbandry

We collected segments of ten different common toad egg strings from Lake Garancsi (47°37′25”N, 18°48′27″E) located in the Pilis Hills, Hungary, in early March 2016, and transported them to a laboratory of the Plant Protection Institute (PPI), Centre for Agricultural Research (CAR), Hungarian Academy of Sciences (HAS). Each egg string was kept separately until the embryos hatched. Two days after hatchlings reached the free-swimming state, we placed tadpoles individually into 2-L rearing containers filled with 0.7 L reconstituted soft water (RSW; APHA 1985). Ambient temperature was set to 21 °C during daylight hours, which was allowed to decrease to 18 °C at night. Lighting was set to a 11:13 dark:light cycle. Tadpoles were fed ad libitum twice a week with a 1:100 mixture of finely ground *Spirulina* (NaturPiac, Budapest, Hungary) and slightly boiled spinach. We changed water in the rearing containers every three days taking care to minimize disturbance to the animals: we carefully drained 90% of the water from the containers with a plastic pipe and refilled them with fresh RSW. All procedures involving animals in this study were approved by the national authority of the Middle-Danube-Valley Inspectorate for Environmental Protection, Nature Conservation and Water Management, Hungary (KTF: 3596–7/2016) and the Ethical Commission of the PPI CAR HAS. All applicable international, national, and institutional guidelines for the care and use of animals were followed.

### Experimental Design and Data Collection

During the setup of the experiment, we applied a randomized full-factorial block design with two factors: treatment (4 levels) and sampling (time since the application of treatments; 7 levels). We haphazardly allocated one tadpole from each of the 10 families to each treatment combination (280 tadpoles). We raised an additional tadpole from each family (10 tadpoles) and preserved it immediately before the treatments were applied to be able to determine initial bufadienolide quantities. We arranged all specimens of each family into a spatial block each, resulting in ten horizontal blocks in the vertical space of the laboratory shelves.

On the 14th day of the experiment, when tadpoles reached Gosner stage 35 (Gosner 1960), individuals were exposed to one of four different treatments: administration of norepinephrine, stressing, only handling and no disturbance. For the determination of initial bufadienolide amounts in tadpoles in baseline conditions, one tadpole from each family was fixed in 70% methanol prior to the application of treatments. To induce the depletion of bufadienolide stores in the tadpoles’ skin, we applied *in vivo* stimulation via hormonal treatment (administration of norepinephrine). Tadpoles in the norepinephrine treatment group were placed into a 3-ml, 100-μM norepinephrine-bitartrate (CAS 3414-63-9, Sigma-Aldrich, USA) solution for 15 min (Kurali et al. 2016; Maag et al. 2012). To facilitate the washing of norepinephrine and any toxins that had been released from the skin, we subsequently transferred individuals into a box filled with 700 ml RSW for one minute and finally placed them back into their rearing containers. For stressing tadpoles, we abruptly prodded them with a blunt glass rod while taking care not to damage the skin of treated animals; this was done only once and without removing animals from the rearing container. In the treatment group receiving only handling, we placed tadpoles into 3 ml of RSW and otherwise handled them the same way as those in the norepinephrine treatment group. Animals in the no disturbance group were left undisturbed throughout the experiment. Zero, 12, 24, 48, 72, 96, and 120 h after applying treatments, we haphazardly selected one tadpole from each family in each treatment (40 tadpoles/sampling) and fixed it in 70% methanol. The resulting sample size was similar to those of previous studies on bufadienolide production in common toad tadpoles (e.g. Bókony et al. 2017; Üveges et al. 2017).

### Chemical Analysis

The collected samples were processed in the Department of Pathophysiology at the PPI, CAR, HAS. We homogenized tadpoles using a VWR VDI 12 homogenizer with an IKA S12 N-7S dispersing tool. After homogenization, samples were dried under vacuum at 45 °C using a rotary evaporator (Büchi Rotavapor R-134, Flawil, Switzerland), and dry mass measured to the nearest 0.0001 g using an Ohaus Pioneer PA-114 analytical balance (Ohaus Corp., Parsippany, NJ, USA). We re-dissolved the samples in 1 ml HPLC grade methanol, which was aided by brief use of ultrasound in a bath sonicator (Tesla UC005AJ1). Finally, we filtered samples using FilterBio nylon syringe filters (pore size = 0.22 μm). We identified toxin compounds as bufadienolides by inspecting the UV spectrum of peaks and by using commercially acquired bufalin, bufotalin, (resi)bufogenin, gamabufotalin, areno- and telocinobufagin (Biopurify Phytochemicals, Chengdu, China), cinobufagin (Chembest, Shanghai, China), cinobufotalin (Quality Phytochemicals, New Jersey, USA), digitoxigenin (Santa Cruz Biotechnology, Dallas, TX, USA) and marinobufotoxin (a courtesy of Prof. Rob J. Capon, Institute for Molecular Bioscience, University of Queensland, Australia) as standards. To identify and quantify the bufadienolide compounds in tadpoles, we used a high-performance liquid chromatography – mass spectrometry (HPLC-MS) system (Shimadzu LC-MS-2020, Shimadzu Corp., Kyoto, Japan) equipped with a binary gradient solvent pump, a vacuum degasser, a thermostated autosampler, a column oven, a diode array detector and a single-quadrupole mass analyser with electrospray ionization (ESI-MS). Chromatographic separations were carried out at 35 °C on a C18 2.6 μm column (Kinetex, 100 mm × 3 mm i.d.) in series with a C18 guard column (4 mm × 3 mm i.d.) using 10 μl injections. The mobile phase consisted of 5% aqueouos acetonitrile containing 0.05% formic acid (solvent A) and acetonitrile containing 0.05% formic acid (solvent B). The flow rate was 0.8 mL/min and the gradient was as follows: 0–2 min, 10.5–21.1% B; 2–15 min, 21.1–26.3% B; 15–24 min, 26.3–47.4% B; 24–25 min, 47.4–100% B; 25–30 min 100% B; 30–31 min 100–10.5% B; 31–35 min 10.5% B. ESI worked under the following conditions: desolvation line (DL) temperature: 250 °C; heat block temperature: 400 °C; drying N_2_ gas flow:15 l min^−1^; nebulizer N_2_ gas flow: 1.5 l min^−1^; positive ionization mode. We acquired and processed the data using the programme LabSolutions 5.42v (Shimadzu Corp.). The above optimization settings have been successfully used to identify and quantify bufadienolide compounds in previous studies (e.g. Bókony et al. 2016; Üveges et al. 2017).

### Statistical Analyses

For calculating the number of bufadienolide compounds (NBC) present in each tadpole, we scored a compound to be present if it was detectable (the limit of detection was between 0.02 and 0.05 ng for the standards) in the chromatogram. The quantity of each compound was estimated from the area under the chromatographic peaks using the calibration curve of the bufotalin standard; these bufotalin-equivalent quantities were subsequently used in the statistical analysis. Both outcomes were measured by an investigator who was blinded to the group allocation during the experiment. We also calculated total bufadienolide quantity (TBQ) for each animal by summing the estimated quantities of each compound (for a similar approach see e.g., Benard and Fordyce 2003; Bókony et al. 2018; Hagman et al. 2009).

We fitted quasi-Poisson generalized linear mixed-effect model (GLMM) using penalized quasi-likelihood estimation to analyse the effects of treatment and sampling on NBC using the ‘MASS’ R package 2002). We used linear mixed-effect models (LMM) to analyse the effects of treatment and different sampling occasions on toxin quantities using the ‘nlme’ R package (Pinheiro et al. 2017). Into these latter models, we included TBQ or the quantity of a single compound as the dependent variable. We applied square-root transformation in the case of TBQ and seven compounds, and log transformation in the case of one compound to improve residuals’ fit to a normal distribution. In the case of another four components residual diagnostics of the initial models indicated insufficient fit due to their highly skewed distributions; here we entered rank-transformed quantities as the dependent variable (Table S4). Three compounds were absent in more than 50% of the tested individuals; so we refrained from analysing these components separately, but took them into account in the calculation of TBQ. We entered treatment and sampling occasion as fixed factors and their interaction into all fitted models, and also added dry weight of each tadpole (mean ± SD: 20.27 ± 6.6 mg) as a potential confounding variable. Deviation from the median NBC was also included as a covariate into the model fitted on TBQ to control for consistent differences between individuals in the number of compounds. Family was incorporated into all models as a random factor, and we allowed heterogeneous variance between treatments in the LMMs by adding the ‘weights’ parameter with ‘varIdent’ to the models. We used a backward elimination procedure for model selection, starting with fitting the full model first, then dropping the predictor with the highest *P*-value in each step and retaining only statistically significant effects (*P* ≤ 0.05) in the final models (Engqvist 2005; Grafen and Hails 2002). Test statistics (type III Wald *χ*^2^) and associated *P*-values were computed using the ‘Anova’ function in the ‘car’ R package (Fox and Weisberg 2011). The ‘weights’ variable was excluded from the model if it had negligible effect on model fit based on a likelihood ratio test; fulfilment of the requirements of the fitted LMMs was checked by plot diagnosis. Post hoc tests with Tukey adjustment were used for pairwise comparisons between treatments within each sampling occasion using the ‘lsmeans’ R package (Lenth 2016). We run all analyses in R 3.4.1 (R Core Team 2017). All tests were two-tailed with α set to 0.05. Data used in the statistical analyses are available from Figshare (https://figshare.com/s/0f4c347e1e4c3f412f92).

## Results

In total, we found 21 putative bufadienolide compounds in the toad tadpoles, of which three could be unambiguously identified as bufotalin, arenobufagin and marinobufotoxin, respectively (Table S1).

The number of bufadienolide compounds (overall median: 18 compounds/tadpole, range: 13–21) was significantly affected by sampling occasions (*χ*^2^_6_ = 35.98, *P* < 0.001, Table 1), but not by the applied treatment (either by itself or in interaction with sampling; both *P* ≥ 0.502, Table 1). Immediately after treatments (0 h), the number of bufadienolide compounds was significantly lower compared to the third (24 h) sampling, and was also lower than on other sampling occasions except for the second (12 h) and fourth (48 h) samplings; for further details see Table S2. The number of bufadienolide compounds also increased slightly, but significantly with tadpoles’ dry mass (*β* ± SE = 0.002 ± 0.0007, Table 1).

**Table 1:** Test statistics and significance of the investigated explanatory variables from the fitted models on the number of bufadienolide compounds (NBC) and total bufadienolide quantity (TBQ)

Dependent variable	Model type	Random effect (family)	Explanatory variables	*χ* ^2^	df	*P*-value
**Number of bufadienolide compounds (NBC)**	**quasi-Poisson GLMM with PQL estimation**	**0.04 [0.03–0.07]**	**Intercept**	**14,759.07**	**1**	**<0.001**
			**Dry mass**	**8.49**	**1**	**0.004**
			**Sampling**	**35.98**	**6**	**<0.001**
			Treatment	1.75	3	0.626
			Sampling × Treatment	17.31	18	0.502
**Total bufadienolide quantity (TBQ)** ^a^	**LMM**	**12.99 [8.01–21.06]**	**Intercept**	**370.23**	**1**	**<0.001**
			**Deviation from the median NBC**	**9.58**	**1**	**0.002**
			**Sampling**	**36.51**	**6**	**<0.001**
			**Treatment**	**22.21**	**3**	**<0.001**
			**Sampling × Treatment**	**33.18**	**18**	**0.016**
			Dry mass	0.75	1	0.388

Final models are shown in bold; test statistics and *P*-values for the non-significant predictors were computed by including them one by one into the final models. Random effects are given in SD ± 95% confidence interval

^a^square-root transformed

**Fig. 1:**
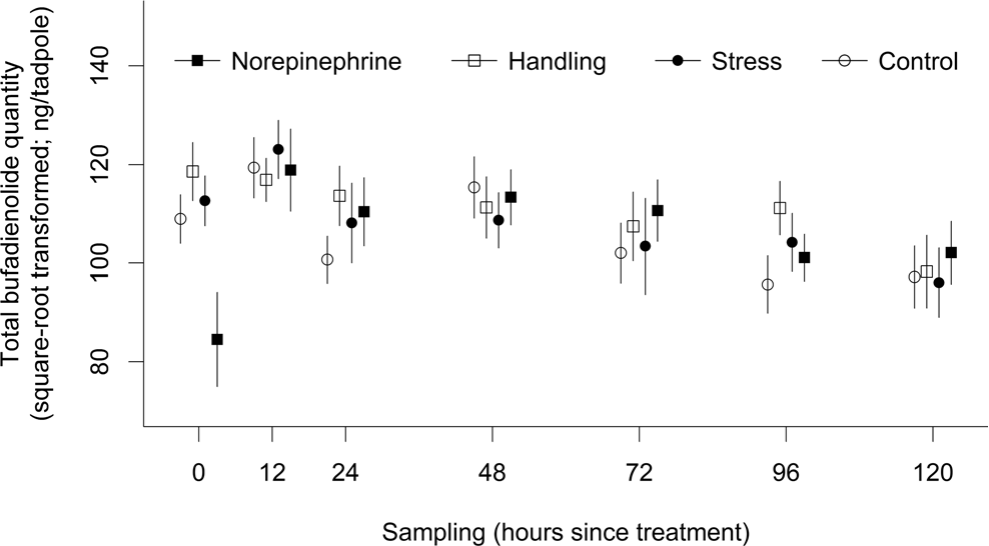
Mean of the total bufadienolide quantity (TBQ) at various sampling occasions. The error bar indicates SE calculated from biological replicates (*n* = 10 tadpoles in each treatment-sampling combination). Tukey post hoc test performed within each sampling occasion showed that immediately after the application of treatments (0 h) TBQ was significantly lower than in any of the other treatments (all *P* ≤ 0.003; see also in Table 2)

Total bufadienolide quantity (overall mean ± SD: 12040.54 ± 4697.37 bufotalin-equivalent ng/tadpole) was significantly affected by the interaction of treatment and sampling (*χ*^2^_18_ = 33.18, *P* = 0.016; Table 1, Fig. 1): immediately after the application of treatments, TBQ was lower in tadpoles that were immersed into norepinephrine solution compared to individuals in other treatments, but this difference disappeared by the second sampling occasion (i.e. after 12 h; Table 2). Total bufadienolide quaility was also lower in the control than in the handling treatment group at the third (24 h) and the sixth (96 h) samplings. All other pairwise comparisons were non-significant (all *P* ≥ 0.400, Table 2). Deviation from the median number of compounds was positively related to TBQ (*β* ± SE = 2.27 ± 0.73, Table 1), suggesting that individuals which produced a greater diversity of compounds had overall greater quantities of bufadienolides, whereas dry mass had no significant effect on TBQ (Table 1).

**Table 2:** Pairwise comparisons of the different treatments within each sampling occasion from the LMM fitted on TBQ

Sampling	Contrast	Estimate	s.e.m.	df	*t* ratio	*P*-value
0 hr	Control - Handling	−7.83	5.78	242	−1.36	0.528
	**Control - Norepinephrine**	**25.09**	**7.07**	**242**	**3.55**	**0.003**
	Control - Stress	−2.54	6.43	242	−0.40	0.979
	**Handling - Norepinephrine**	**32.93**	**7.15**	**242**	**4.61**	**<0.001**
	Handling - Stress	5.29	6.50	242	0.81	0.848
	**Norepinephrine - Stress**	**−27.64**	**7.69**	**242**	**−3.59**	**0.002**
12 hrs	Control - Handling	1.12	5.76	242	0.19	0.997
	Control - Norepinephrine	−1.34	7.10	242	−0.19	0.998
	Control - Stress	−3.94	6.42	242	−0.61	0.927
	Handling - Norepinephrine	−2.46	7.14	242	−0.34	0.986
	Handling - Stress	−5.06	6.50	242	−0.78	0.864
	Norepinephrine - Stress	−2.60	7.71	242	−0.34	0.987
24 hrs	**Control - Handling**	**−15.69**	**5.81**	**242**	**−2.70**	**0.037**
	Control - Norepinephrine	−11.10	7.08	242	−1.57	0.400
	Control - Stress	−8.12	6.42	242	−1.26	0.586
	Handling - Norepinephrine	4.59	7.15	242	0.64	0.918
	Handling - Stress	7.57	6.53	242	1.16	0.653
	Norepinephrine - Stress	2.97	7.69	242	0.39	0.980
48 hrs	Control - Handling	2.48	5.77	242	0.43	0.973
	Control - Norepinephrine	2.00	7.07	242	0.28	0.992
	Control - Stress	6.21	6.42	242	0.97	0.768
	Handling - Norepinephrine	−0.49	7.16	242	−0.07	1.000
	Handling - Stress	3.73	6.50	242	0.57	0.940
	Norepinephrine - Stress	4.21	7.69	242	0.55	0.947
72 hrs	Control - Handling	−6.08	5.75	242	−1.06	0.716
	Control - Norepinephrine	−9.75	7.08	242	−1.38	0.515
	Control - Stress	−3.86	6.47	242	−0.60	0.933
	Handling - Norepinephrine	−3.67	7.14	242	−0.51	0.956
	Handling - Stress	2.22	6.52	242	0.34	0.986
	Norepinephrine - Stress	5.89	7.70	242	0.76	0.870
96 hrs	**Control - Handling**	**−15.69**	**5.75**	**242**	**−2.73**	**0.034**
	Control - Norepinephrine	−4.72	7.07	242	−0.67	0.909
	Control - Stress	−8.03	6.42	242	−1.25	0.595
	Handling - Norepinephrine	10.96	7.14	242	1.53	0.418
	Handling - Stress	7.65	6.50	242	1.18	0.641
	Norepinephrine - Stress	−3.31	7.69	242	−0.43	0.973
120 hrs	Control - Handling	−1.32	5.75	242	−0.23	0.996
	Control - Norepinephrine	−5.14	7.07	242	−0.73	0.886
	Control - Stress	−0.23	6.43	242	−0.04	1.000
	Handling - Norepinephrine	−3.82	7.14	242	−0.54	0.950
	Handling - Stress	1.09	6.50	242	0.17	0.998
	Norepinephrine - Stress	4.91	7.70	242	0.64	0.920

*P*-values were calculated using tukey HSD adjustment; estimates and SE values are expressed on the transformed (Square-Root) scale. Significant contrasts are shown in bold

Out of the 18 individually analysed bufadienolides, the quantity of 11 compounds was significantly affected by the interaction between treatment and the timing of sampling (Table S3). Six of these compounds were characterized by >700 m/z value and their amounts significantly decreased during the hormonal treatment, but were restored after 12 h similarly to TBQ (Table 3). The change in quantities of the other five compounds (all <600 m/z value) showed an opposite pattern, being significantly higher immediately after the norepinephrine treatment than in the control group (the quantity of four compounds was also higher than in the ‘handling’ treatment group, and for three compounds also higher than in the ‘stress’ treatment group), while this difference also disappeared after 12 h (Table 3). The rest of the compounds (seven bufadienolides) varied significantly among sampling occasions, but were not affected by treatment (either by itself or in interaction with sampling; all *P* ≥ 0.066, Table S3). Also, dry mass was positively related to the quantity of bufadienolides in eight out of the 18 compounds (Table S3).

**Table 3:** Estimates and SE values on contrasts of individual bufadienolide compounds’ quantity between the norepinephrine and other treatment groups immediately and 12 hrs after treatment, expressed on the transformed scale

Compound	m/z value	Sampling	Control - Norepinephrine	Handling - Norepinephrine	Norepinephrine - Stress
Unidentified compound 2^a^	367	0 hr	**−5.08 ± 1.03**	**−4.22 ± 1.26**	**3.28 ± 1.21**
		12 hrs	−0.40 **±** 1.03	0.70 **±** 1.25	−1.19 **±** 1.21
Unidentified compound 5^b^	417	0 hr	**−0.60 ± 0.14**	−0.24 **±** 0.15	**0.42 ± 0.15**
		12 hrs	−0.10 **±** 0.14	−0.09 **±** 0.15	−0.04 **±** 0.15
Bufotalin^c^	445	0 hr	**−122.39 ± 26.50**	**−93.81 ± 26.52**	**80.64 ± 26.36**
		12 hrs	−14.38 **±** 26.51	−13.77 **±** 26.28	−7.95 **±** 26.29
Unidentified compound 7^a^	571	0 hr	**−5.54 ± 1.43**	**−4.66 ± 1.4 3**	2.54 **±** 1.42
		12 hrs	−1.11 **±** 1.43	−1.01 **±** 1.41	0.44 **±** 1.41
Unidentified compound 8^a^	573	0 hr	**−4.18 ± 1.17**	**−3.77 ± 1.18**	2.98 **±** 1.41
		12 hrs	0.99 **±** 1.17	0.91 **±** 1.17	−1.27 **±** 1.40
Unidentified compound 11	715	0 hr	**1410.47 ± 447.57**	**1859.34 ± 407.66**	**−1278.42 ± 444.50**
		12 hrs	147.15 **±** 447.57	55.36 **±** 407.66	−443.82 **±** 444.50
Unidentified compound 12^a^	715	0 hr	**9.09 ± 2.52**	**13.04 ± 2.52**	**−8.76 ± 2.52**
		12 hrs	1.97 **±** 2.52	−0.97 **±** 2.52	−4.61 **±** 2.52
Unidentified compound 13	727	0 hr	**691.78 ± 231.26**	**991.83 ± 232.28**	**−762.40 ± 245.46**
		12 hrs	−4.72 **±** 231.26	−199.50 **±** 232.28	−44.17 **±** 245.46
Unidentified compound 14^a^	729	0 hr	**14.84 ± 3.93**	**18.63 ± 4.08**	**−16.42 ± 4.14**
		12 hrs	−0.92 **±** 3.93	−1.96 **±** 4.08	−0.70 **±** 4.14
Unidentified compound 15	729	0 hr	**241.44 ± 65.29**	**312.13 ± 58.88**	**−300.42 ± 70.56**
		12 hrs	−54.86 **±** 65.29	−97.59 **±** 58.88	103.55 **±** 70.56
Unidentified compound 18	757	0 hr	**967.25 ± 289.62**	**1394.10 ± 294.01**	**−1220.98 ± 313.10**
		12 hrs	−67.91 **±** 289.62	−118.22 **±** 294.01	−92.87 **±** 313.10

Only compounds with amounts that were significantly affected by the sampling × treatment interaction in the fitted lmms are included in the Table. *P*-values were calculated using tukey HSD tests; significant contrasts are shown in bold

^a^square-root transformed

^b^log-transformed

^c^rank-transformed

## Discussion

Our results provide experimental evidence for fast changes in the quantities of bufadienolides in common toad tadpoles. Total bufadienolide quantity was significantly lower in treated compared to untreated tadpoles immediately after stimulating bufadienolide release induced by immersion of treated individuals into norepinephrine solution, but it become similar in treated and untreated tadpoles within only 12 h. We observed this change only in hormone-treated individuals, but not in tadpoles from other treatment groups, which suggests that animals did not secrete bufadienolides onto their skin surface in response to stress stimuli. Interestingly, some compounds showed opposite changes in their quantity following induced bufadienolide release, while the amount of six out of ten bufadienolides characterized by >700 m/z values (most probably conjugate-type bufotoxins) decreased significantly, five out of 11 bufadienolides characterized by <600 m/z values (most likely free-type bufogenins) had higher quantities immediately after the applied hormonal treatment. The rest of the compounds were either not affected by the norepinephrine treatment (these could be present only in other tissues of the tadpoles; Matsukawa et al. 1996) or were not individually analysed due to their absence in more than half of the tadpoles. Nevertheless, after 12 h all examined bufadienolides were present in similar amounts as in the control. The number of bufadienolide compounds was, in accordance with our initial prediction, not affected by the applied treatments. In the following sections we provide possible explanations for this pattern and discuss the ecological relevance of our findings in the context of chemical defence.

We found that none of the 700 m/z bufadienolides showed an immediate increase in their quantity and none of the <600 m/z compounds had an immediate decrease in their amount in response to the norepinephrine stimulus, which implies a structure-related difference in these components’ role in the bufadienolide expression pathway. If free-type bufogenins are indeed more potent agents of toxicity in common toads (Meng et al. 2016; but see Shimada et al. 1985, 1986, 1987b), animals may store bufadienolides in the form of conjugated bufotoxins to facilitate their *in situ* transport and avoid self-poisoning during storage. Upon secretion, these compounds may be transformed into active bufogenins through enzymatic catalysis (either by their own specific enzymes or through bacterial biotransformation; Hayes et al. 2009; Kamalakkannan et al. 2017), to elicit a strong deterrent/toxic effect once shed onto the surface of the skin. As potent toxins are usually more lipophilic (enabling them to be transported by lipoproteins with blood and to be absorbed swiftly through lipid membranes; Dekant 2009), these compounds can provide protection to the producing individuals until the secreted toxic blend eventually washes off. This idea is supported by the fact that in baseline conditions <600 m/z compounds were present in much lower quantities in the toxin blend than >700 m/z bufadienolides in the tadpoles (mean ± SD: 562.2 ± 207.7 vs. 11,032.6 ± 4604.3 bufotalin-equivalent ng/tadpole; Wilcoxon signed rank test: *V* = 55, *n* = 10, *P* = 0.002). Alternatively, bufadienolides characterized by lower m/z values are, in fact, precursors of the stored, conjugated bufotoxins, and their quantity increased in our experiment because of rapid *de novo* synthesis during the applied hormonal treatment. In this case, both the biosynthesis of bufogenins and dissolution of bufotoxins into the water from the tadpoles’ skin surface took place within a mere 15 min during the experiment. Previous studies of experimentally induced toxin release in toads usually applied more invasive methods, were conducted on juveniles or adults in a much coarser time scale, and toxin replenishment inside the parotoid glands’ alveoli was only inferred, not explicitly quantified (e.g. Jared et al. 2009, 2014; Toledo et al. 1992). Thus, as long as we do not have more detailed information about the bufadienolide expression pathway in bufonids, further investigations on potential differences in toxicity and water solubility among these toxin compounds and on differences in their proportion in the secreted and stored toxin blend may indirectly support one of these alternative explanations.

We found that bufadienolide toxin levels became similar surprisingly fast, within 12 h, in the hormone-treated tadpoles compared to their control counterparts. Amphibian toxin glands are typically specialized for passive defence (Jared et al. 2009; Mailho-Fontana et al. 2014), and toxin release is induced by a sufficiently high external pressure (e.g. during predator attack), which may be also true for the much more simply structured unicellular skin glands in tadpoles (as also suggested in Delfino et al. 1995, p. 111). This may also explain why the total bufadienolide quantity changed only in the hormone-stimulated tadpoles in our experiment and not in response to physical disturbance caused by the handling procedure or the applied stress stimulus. The rapid offset of toxin reserves in treated and untreated tadpoles may indicate relatively relaxed resource requirements for bufadienolide production, which is consistent with our previous work, where we found that toxin release induced multiple times during larval development had low fitness costs in common toad tadpoles (Kurali et al. 2016). These findings indicate that common toad tadpoles are chemically well-defended at almost all times against recurring attacks of predators that are susceptible to bufadienolide toxins, and individuals can maintain their toxicity without considerable trade-offs with other predator-induced traits. However, this may only be true if such attacks do not frequently result in injuries, which can substantially alter the fitness costs associated with passive chemical defence, i.e. if toxins are secreted and become effective only once prey are attacked and grabbed by the predator. In a recent meta-analysis, Zvereva and Kozlov (2016) also found no detectable physiological costs of the production of chemical defence against predators in herbivorous insects, and concluded that ecological costs in those species may be more important in trade-offs associated with chemical defence than the costs of acquiring the necessary resources.

While we still have limited information about many aspects of the bufadienolide-based chemical defence in bufonids, other defensive toxins have been investigated in more detail both in amphibians and in other organisms. For example, many marine and terrestrial animals, like echinoderms, arthropods, gastropods, fish and amphibians are known to contain tetrodotoxin (TTX), a highly potent non-proteinaceous neurotoxin, as a chemical defence against predators (reviewed in Magarlamov et al. 2017). Although in many TTX-bearing organisms this compound is acquired either through dietary uptake or via symbiosis with TTX producing bacteria, some terrestrial newts like the rough-skin newt, *Taricha granulosa*, may be able to produce this toxin *de novo* (Gall et al. 2014). Previous findings in this species revealed that individual TTX concentrations show considerable fluctuation in newt populations in the wild (Bucciarelli et al. 2016), and population variation in TTX concentrations may, at least partly, be the result of a coevolutionary arms race between newts and a predatory snake (Brodie et al. 2002). In laboratory studies, adults were also found to be able to regenerate their toxin following experimentally induced secretion (Cardall et al. 2004) and to rapidly invest into toxin production as a result of simulated failed predator attack similarly to their larvae (Bucciarelli et al. 2017).

In conclusion, we showed that the significant decrease in total bufadienolide quantity induced by an experimentally stimulated toxin release vanished within 12 h in common toad tadpoles. We also uncovered a remarkable difference in the temporal dynamics of some bufadienolides within the toxin blend, emphasizing a different role of toxin compounds in tadpoles’ chemical defence depending on their structural complexity. Further studies are needed to clarify the functional differences between various bufadienolide compounds and the exact proximate mechanism of toxin expression in bufonids, one of the most important vertebrate model organisms of inducible chemical defence.

## Electronic supplementary material

ESM 1 (DOCX 196 kb)
