# Supplementary material for: Changes in Toxin Quantities Following Experimental Manipulation of Toxin Reserves in Bufo bufo Tadpoles
Source: J Chem Ecol. 2019 Jan 26;45(3):253–63. doi: 10.1007/s10886-019-01045-9 (PMC6477007; doi:10.1007/s10886-019-01045-9)
Supplement: Supplementary file 1 — (DOCX 196 kb) [file 10886_2019_1045_MOESM1_ESM.docx]

Electronic Supplementary Material

CHANGES in Toxin quantities Following Experimental manipulation of Toxin Reserves in *Bufo bufo* Tadpoles

Submitted to Journal of Chemical Ecology

Zoltán Tóth^1*^, Anikó Kurali^1^, Ágnes M. Móricz^2^, Attila Hettyey^1^

*^1^Lendület Evolutionary Ecology Research Group, Plant Protection Institute, Centre for Agricultural Research, Hungarian Academy of Sciences, Herman Ottó Str. 15, H-1022 Budapest, Hungary*

*^2^Department of Pathophysiology, Plant Protection Institute, Centre for Agricultural Research, Hungarian Academy of Sciences, Herman Ottó Str. 15, H-1022 Budapest, Hungary*

*Corresponding author. Z. Tóth, E-mail: [toth.zoltan@agrar.mta.hu](mailto:toth.zoltan@agrar.mta.hu), Phone: +36-1-3918609

Table S1. Mass signal and retention time of the detected bufadienolide compounds with their percent occurrence in common toad tadpoles before and after treatment

| Compound name | m/z value in positive ionization mode [M+H]^+^ | Retention time (min) | Pre-treatment percent occurrence  (*n* = 10) | Post-treatment percent occurrence  (*n* = 280) |
| --- | --- | --- | --- | --- |
| Unidentified compound 1 | 365 | 23.5 | 20 | 18.93 |
| Unidentified compound 2 | 367 | 22.2 | 100 | 97.14 |
| Unidentified compound 3 | 401 | 11.98 | 0 | 6.79 |
| Unidentified compound 4  (Bufotalinin)^a^ | 415 | 6.44 | 100 | 78.93 |
| Arenobufagin | 417 | 4.756 | 10 | 45.71 |
| Unidentified compound 5 | 417 | 5.236 | 100 | 100 |
| Unidentified compound 6 | 433 | 2.56 | 100 | 88.21 |
| Bufotalin | 445 | 10.2 | 50 | 74.29 |
| Unidentified compound 7 | 571 | 20.78 | 100 | 99.29 |
| Unidentified compound 8 | 573 | 19.5 | 60 | 94.64 |
| Unidentified compound 9 | 601 | 25.1 | 80 | 80.36 |
| Marinobufotoxin | 713 | 12.2 | 100 | 99.64 |
| Unidentified compound 10 | 713 | 17.7 | 100 | 100 |
| Unidentified compound 11 | 715 | 10.5 | 100 | 100 |
| Unidentified compound 12 | 715 | 13.5 | 100 | 98.57 |
| Unidentified compound 13 | 727 | 7.4 | 100 | 100 |
| Unidentified compound 14 | 729 | 6.8 | 100 | 100 |
| Unidentified compound 15 | 729 | 9.2 | 100 | 99.64 |
| Unidentified compound 16 | 729 | 9.9 | 100 | 100 |
| Unidentified compound 17 | 731 | 5.8 | 100 | 99.29 |
| Unidentified compound 18  (Bufotoxin)^a^ | 757 | 17 | 100 | 100 |

^a^ tentatively identified based on molar mass using HPLC-MS and MS-MS

**Table S2.** Pairwise comparisons of the different sampling occasions from the GLMM fitted on NBC. *P*-values were calculated using Tukey HSD adjustment; estimates and SE values are expressed on the transformed (log) scale. Significant contrasts are written in bold.

| Contrast | Estimate | SE | df | *t* ratio | *P*-value |
| --- | --- | --- | --- | --- | --- |
| 0 hr vs. 12 hrs | -0.030 | 0.016 | 263 | -1.85 | 0.513 |
| **0 hr vs. 24 hrs** | **-0.074** | **0.016** | **263** | **-4.53** | **<0.001** |
| 0 hr vs. 48 hrs | -0.017 | 0.016 | 263 | -1.05 | 0.941 |
| **0 hr vs. 72 hrs** | **-0.053** | **0.016** | **263** | **-3.31** | **0.018** |
| **0 hr vs. 96 hrs** | **-0.069** | **0.016** | **263** | **-4.28** | **0.001** |
| **0 hr vs. 120 hrs** | **-0.055** | **0.016** | **263** | **-3.42** | **0.013** |
| 12 hrs vs. 24 hrs | -0.044 | 0.016 | 263 | -2.72 | 0.097 |
| 12 hrs vs. 48 hrs | 0.013 | 0.016 | 263 | 0.81 | 0.984 |
| 12 hrs vs. 72 hrs | -0.023 | 0.016 | 263 | -1.42 | 0.788 |
| 12 hrs vs. 96 hrs | -0.038 | 0.016 | 263 | -2.39 | 0.207 |
| 12 hrs vs. 120 hrs | -0.025 | 0.016 | 263 | -1.53 | 0.729 |
| **24 hrs vs. 48 hrs** | **0.057** | **0.016** | **263** | **3.51** | **0.009** |
| 24 hrs vs. 72 hrs | 0.021 | 0.016 | 263 | 1.28 | 0.860 |
| 24 hrs vs. 96 hrs | 0.005 | 0.016 | 263 | 0.32 | 1 |
| 24 hrs vs. 120 hrs | 0.019 | 0.016 | 263 | 1.16 | 0.908 |
| 48 hrs vs. 72 hrs | -0.036 | 0.016 | 263 | -2.25 | 0.272 |
| **48 hrs vs. 96 hrs** | **-0.052** | **0.016** | **263** | **-3.22** | **0.024** |
| 48 hrs vs. 120 hrs | -0.038 | 0.016 | 263 | -2.36 | 0.219 |
| 72 hrs vs. 96 hrs | -0.015 | 0.016 | 263 | -0.97 | 0.960 |
| 72 hrs vs. 120 hrs | -0.002 | 0.016 | 263 | -0.11 | 1 |
| 96 hrs vs. 120 hrs | 0.014 | 0.016 | 263 | 0.86 | 0.978 |

**Table S3.** Test statistics and significance of the investigated explanatory variables from the LMMs fitted on the amount of individual bufadienolide compounds. Arenobufagin and unidentified compounds 1 and 3 were not analysed individually, and therefore were excluded from the table. Final models are shown in bold; test statistics and *P*-values for the non-significant predictors were computed by including them one by one into the final models. Random effects are given in SD ± 95% confidence interval.

| Compound | Transformation | Random effect (family) | Explanatory variables | *χ*^2^ | df | *P*-value |
| --- | --- | --- | --- | --- | --- | --- |
| Bufotalin | rank(x) | 45.82 [28.10-74.73] | **Intercept** | **0.02** | **1** | **0.887** |
|  |  |  | **Dry mass** | **21.15** | **1** | **<0.001** |
|  |  |  | **Sampling** | **46.01** | **6** | **<0.001** |
|  |  |  | **Treatment** | **23.39** | **3** | **<0.001** |
|  |  |  | **Sampling × Treatment** | **35.43** | **18** | **0.008** |
| Marinobufotoxin | rank(x) | 55.32 [34.19-89.51] | **Intercept** | **48.95** | **1** | **<0.001** |
|  |  |  | **Sampling** | **23.17** | **6** | **0.001** |
|  |  |  | Dry mass | 0.02 | 1 | 0.879 |
|  |  |  | Treatment | 5.95 | 3 | 0.114 |
|  |  |  | Sampling × Treatment | 27.76 | 18 | 0.066 |
| Unidentified compound 2 | sqrt(x) | 1.26 [0.74-2.14] | **Intercept** | **54.55** | **1** | **<0.001** |
|  |  |  | **Dry mass** | **25.57** | **1** | **<0.001** |
|  |  |  | **Sampling** | **23.57** | **6** | **0.001** |
|  |  |  | **Treatment** | **25.34** | **3** | **<0.001** |
|  |  |  | **Sampling × Treatment** | **50.26** | **18** | **<0.001** |
| Unidentified compound 4 | rank(x) | 30.33 [17.54-52.46] | **Intercept** | **46.15** | **1** | **<0.001** |
|  |  |  | **Dry mass** | **6.22** | **1** | **0.013** |
|  |  |  | **Sampling** | **64.89** | **6** | **<0.001** |
|  |  |  | Treatment | 1.05 | 3 | 0.789 |
|  |  |  | Sampling × Treatment | 22.91 | 18 | 0.194 |
| Unidentified compound 5 | log(x) | 0.24 [0.14-0.38] | **Intercept** | **557.85** | **1** | **<0.001** |
|  |  |  | **Dry mass** | **7.21** | **1** | **0.007** |
|  |  |  | **Sampling** | **34.53** | **6** | **<0.001** |
|  |  |  | **Treatment** | **20.06** | **3** | **<0.001** |
|  |  |  | **Sampling × Treatment** | **39.66** | **18** | **0.002** |
| Unidentified compound 6 | - | 48.42 [29.94-78.32] | **Intercept** | **14.88** | **1** | **<0.001** |
|  |  |  | **Sampling** | **25.24** | **6** | **<0.001** |
|  |  |  | Dry mass | 0.92 | 1 | 0.338 |
|  |  |  | Treatment | 2.61 | 3 | 0.455 |
|  |  |  | Sampling × Treatment | 17.04 | 18 | 0.520 |
| Unidentified compound 7 | sqrt(x) | 1.57 [0.93-2.68] | **Intercept** | **18.77** | **1** | **<0.001** |
|  |  |  | **Dry mass** | **28.44** | **1** | **<0.001** |
|  |  |  | **Sampling** | **27.58** | **6** | **<0.001** |
|  |  |  | **Treatment** | **17.90** | **3** | **<0.001** |
|  |  |  | **Sampling × Treatment** | **39.74** | **18** | **0.002** |
| Unidentified compound 8 | sqrt(x) | 0.33 [0.19-0.59] | **Intercept** | **15.16** | **1** | **<0.001** |
|  |  |  | **Dry mass** | **15.98** | **1** | **<0.001** |
|  |  |  | **Sampling** | **50.81** | **6** | **<0.001** |
|  |  |  | **Treatment** | **14.20** | **3** | **0.003** |
|  |  |  | **Sampling × Treatment** | **38.20** | **18** | **0.004** |
| Unidentified compound 9 | rank(x) | 34.82 [20.85-58.15] | **Intercept** | **4.57** | **1** | **0.033** |
|  |  |  | **Dry mass** | **18.49** | **1** | **<0.001** |
|  |  |  | **Sampling** | **102.35** | **6** | **<0.001** |
|  |  |  | Treatment | 3.33 | 3 | 0.344 |
|  |  |  | Sampling × Treatment | 26.98 | 18 | 0.079 |
| Unidentified compound 10 | - | 27.34 [16.72-44.71] | **Intercept** | **331.96** | **1** | **<0.001** |
|  |  |  | **Sampling** | **100.28** | **6** | **<0.001** |
|  |  |  | Dry mass | 3.10 | 1 | 0.078 |
|  |  |  | Treatment | 3.45 | 3 | 0.328 |
|  |  |  | Sampling × Treatment | 15.34 | 18 | 0.638 |
| Unidentified compound 11 | - | 1203.51 [751.18-1928.19] | **Intercept** | **38.13** | **1** | **<0.001** |
|  |  |  | **Sampling** | **19.82** | **6** | **0.003** |
|  |  |  | **Treatment** | **20.97** | **3** | **<0.001** |
|  |  |  | **Sampling × Treatment** | **29.80** | **18** | **0.039** |
|  |  |  | Dry mass | 0.60 | 1 | 0.438 |
| Unidentified compound 12 | sqrt(x) | 5.47 [3.38-8.83] | **Intercept** | **75.53** | **1** | **<0.001** |
|  |  |  | **Sampling** | **6.00** | **6** | **0.423** |
|  |  |  | **Treatment** | **28.54** | **3** | **<0.001** |
|  |  |  | **Sampling × Treatment** | **37.84** | **18** | **0.004** |
|  |  |  | Dry mass | 1.97 | 1 | 0.160 |
| Unidentified compound 13 | - | 392.16 [241.34-637.25] | **Intercept** | **64.61** | **1** | **<0.001** |
|  |  |  | **Sampling** | **41.00** | **6** | **<0.001** |
|  |  |  | **Treatment** | **18.48** | **3** | **<0.001** |
|  |  |  | **Sampling × Treatment** | **30.92** | **18** | **0.029** |
|  |  |  | Dry mass | 0.23 | 1 | 0.630 |
| Unidentified compound 14 | sqrt(x) | 6.74 [4.15-10.95] | **Intercept** | **208.48** | **1** | **<0.001** |
|  |  |  | **Sampling** | **24.96** | **6** | **<0.001** |
|  |  |  | **Treatment** | **22.91** | **3** | **<0.001** |
|  |  |  | **Sampling × Treatment** | **31.99** | **18** | **0.022** |
|  |  |  | Dry mass | 0.58 | 1 | 0.448 |
| Unidentified compound 15 | - | 76.36 [45.71-127.56] | **Intercept** | **98.74** | **1** | **<0.001** |
|  |  |  | **Sampling** | **33.72** | **6** | **<0.001** |
|  |  |  | **Treatment** | **30.43** | **3** | **<0.001** |
|  |  |  | **Sampling × Treatment** | **46.11** | **18** | **<0.001** |
|  |  |  | Dry mass | 0.76 | 1 | 0.383 |
| Unidentified compound 16 | - | 36.86 [23.01-59.05] | **Intercept** | **24.46** | **1** | **<0.001** |
|  |  |  | **Dry mass** | **4.56** | **1** | **0.033** |
|  |  |  | **Sampling** | **31.64** | **6** | **<0.001** |
|  |  |  | **Treatment** | **8.33** | **3** | **0.040** |
|  |  |  | Sampling × Treatment | 17.52 | 18 | 0.487 |
| Unidentified compound 17 | sqrt(x) | 8.28 [5.14-13.34] | **Intercept** | **127.53** | **1** | **<0.001** |
|  |  |  | **Sampling** | **21.46** | **6** | **0.002** |
|  |  |  | Dry mass | 1.46 | 1 | 0.227 |
|  |  |  | Treatment | 1.08 | 3 | 0.783 |
|  |  |  | Sampling × Treatment | 26.25 | 18 | 0.094 |
| Unidentified compound 18 | - | 646.48 [401.49-1040.97] | **Intercept** | **69.76** | **1** | **<0.001** |
|  |  |  | **Sampling** | **33.34** | **6** | **<0.001** |
|  |  |  | **Treatment** | **24.17** | **3** | **<0.001** |
|  |  |  | **Sampling × Treatment** | **32.46** | **18** | **0.019** |
|  |  |  | Dry mass | 0.89 | 1 | 0.347 |

Figure S1. Calibration curves of the 10 standards used for the identification of toxin compounds as bufadienolides. Similarly to previous studies (e.g. Bókony et al. 2016, 2017, 2018; Üveges et al. 2017), the quantity of each compound was estimated from the area of the chromatographic peaks using the calibration curve of the bufotalin standard.


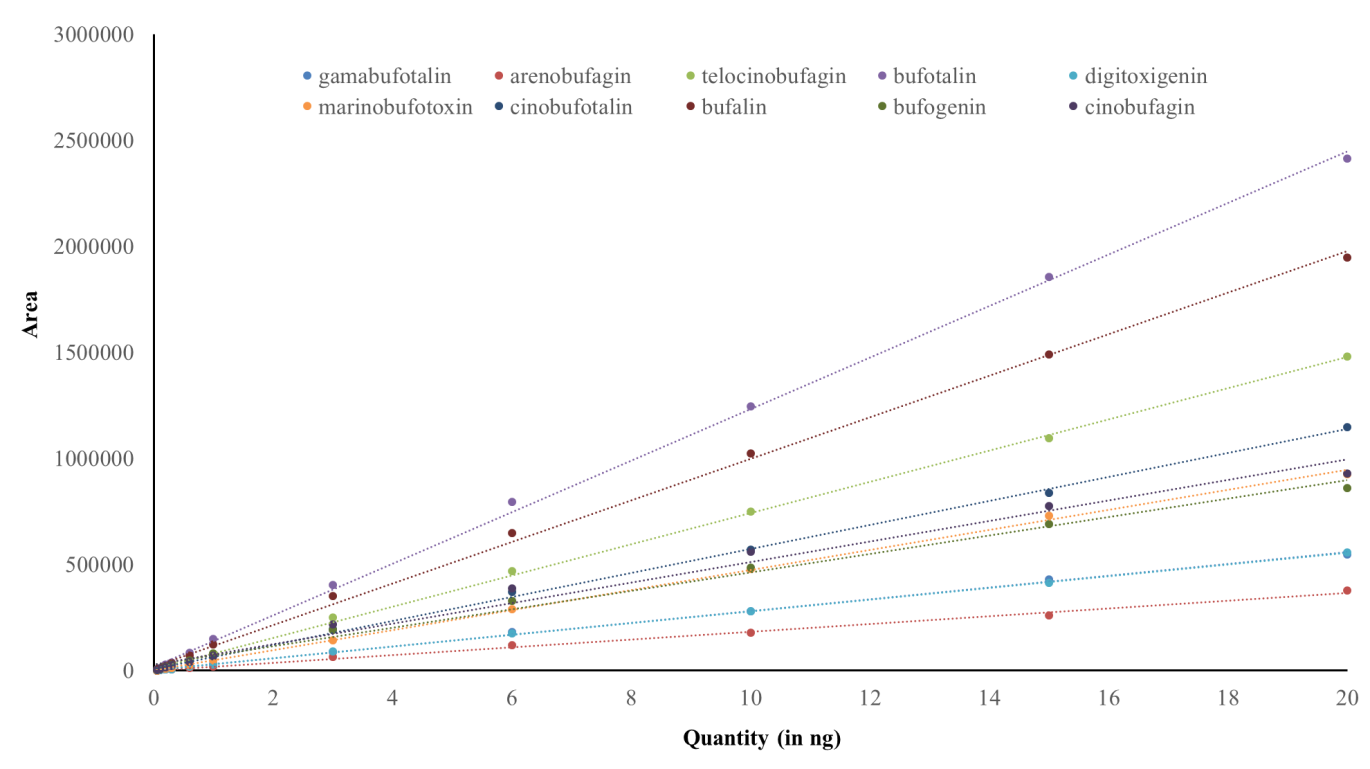


**References**

Bókony V, Móricz ÁM, Tóth Zs, Gál Z, Kurali A, Mikó Zs, Pásztor K, Szederkényi M, Tóth Z, Ujszegi J, Üveges B, Krüzselyi D, Capon RJ, Hoi H, Hettyey A (2016) Variation in chemical defense among natural populations of common toad (*Bufo bufo*) tadpoles: the role of environmental factors. J Chem Ecol 42:329–338.

Bókony V, Mikó Z, Móricz ÁM, Krüzselyi D, Hettyey A (2017) Chronic exposure to a glyphosate-based herbicide makes toad larvae more toxic. Proc R Soc B Biol Sci 284:20170493.

Bókony V, Üveges B, Móricz ÁM, Hettyey A (2018) Competition induces increased toxin production in toad larvae without allelopathic effects on heterospecific tadpoles. Func Ecol 32:667–675.

Üveges B, Fera G, Móricz ÁM, Krüzselyi D, Bókony V, Hettyey A (2017) Age- and environment-dependent changes in chemical defences of larval and post-metamorphic toads. BMC Evol Biol 17:137.
